# Oxidative Damage of Blood Platelets Correlates with the Degree of Psychophysical Disability in Secondary Progressive Multiple Sclerosis

**DOI:** 10.1155/2020/2868014

**Published:** 2020-06-17

**Authors:** Angela Dziedzic, Agnieszka Morel, Elzbieta Miller, Michal Bijak, Tomasz Sliwinski, Ewelina Synowiec, Michal Ceremuga, Joanna Saluk-Bijak

**Affiliations:** ^1^Department of General Biochemistry, Faculty of Biology and Environmental Protection, University of Lodz, Pomorska 41/143, 90-236 Lodz, Poland; ^2^Department of Neurological Rehabilitation, Medical University of Lodz, Milionowa 14, 93-113 Lodz, Poland; ^3^Biohazard Prevention Centre, Faculty of Biology and Environmental Protection, University of Lodz, Pomorska 141/143, 90-236 Lodz, Poland; ^4^Department of Molecular Genetics, Laboratory of Medical Genetics, Faculty of Biology and Environmental Protection, University of Lodz, Pomorska 141/143, Lodz, Poland; ^5^Military Institute of Armament Technology, Prymasa Stefana Wyszyńskiego 7, 05-220 Zielonka, Poland

## Abstract

The results of past research studies show that platelets are one of the main sources of reactive oxygen species (ROS) and reactive nitrogen species (RNS) to be found in the course of many pathological states. The aim of this study was to determine the level of oxidative/nitrative stress biomarkers in blood platelets obtained from multiple sclerosis (MS) patients (*n* = 110) and to verify their correlation with the clinical parameters of the psychophysical disability of patients. The mitochondrial metabolism of platelets was assessed by measuring the intracellular production of ROS using the fluorescence method with DCFH-DA dye and by identification of changes in the mitochondrial membrane potential of platelets using the JC-1 dye. Moreover, we measured the mRNA expression for the gene encoding the cytochrome c oxidase subunit I (*MTCO-1*) and glyceraldehyde 3-phosphate dehydrogenase (*GAPDH*) in platelets and megakaryocytes using the RT-qPCR method, as well as the concentration of NADPH oxidase (NOX-1) by the ELISA method. Our results proved an increased level of oxidative/nitrative damage of proteins (carbonyl groups, 3-nitrotyrosine) (*p* < 0.0001) and decreased level of -SH in MS (*p* < 0.0001) and also a pronounced correlation between these biomarkers and parameters assessed by the Expanded Disability Status Scale and the Beck's Depression Inventory. The application of fluorescence methods showed mitochondrial membrane potential disruption (*p* < 0.001) and higher production of ROS in platelets from MS compared to control (*p* < 0.0001). Our research has also confirmed the impairment of red-ox metabolism in MS, which was achieved by increasing the relative mRNA expression in platelets for the genes studied (2-fold increase for the *MTCO-1* gene and 1.5-fold increase in *GAPDH* gene, *p* < 0.05), as well as the augmented concentration of NOX-1 compared to control (*p* < 0.0001). Our results indicate that the oxidative/nitrative damage of platelets is implicated in the pathophysiology of MS, which reflects the status of the disease.

## 1. Introduction

Oxidative stress plays an important role in regulating brain plasticity, and the intensive production of reactive oxygen species (ROS) and reactive nitrogen species (RNS) significantly affect the disorder of neuronal neurotransmission, which is the main cause of physical and mental disability [1, 2]. Brain structures are prone to oxidative stress due to their extensive oxygen metabolism [3]. Despite the existence of natural antioxidant mechanisms, the cells of the central nervous system (CNS), especially neurons, are poorly protected against the harmful effects of ROS/RNS [4]. ROS and RNS are extremely reactive molecules that damage various cellular structures in the brain (neurons, oligodendrocytes, astrocytes, and microglia) and lead to cell death [5].

Multiple sclerosis (MS) is a multifactorial disease that consists of several pathological processes occurring in the CNS and peripheral nervous system (PNS). MS is an inflammatory demyelinating disease of CNS, nonetheless, tightly related to the injury of blood vessels, mainly as a result of augmented permeability of the blood-brain barrier (BBB) [6, 7]. Autoimmune development, inflammation, and the permanent oxidative stress contribute to demyelination and in consequence to axonal and neuronal loss [8, 9].

The interactions of platelets with leukocytes and endothelial cells are considered to be the first essential step in the initiation of the pathogenesis of MS, leading to the massive infiltration of lymphocytes and further to the creation of demyelinating lesions in CNS [10]. The chronic activation of platelets in MS is proven [11–13], even though their role in this pathology still needs to be clarified. The latest clinical reports confirm an increased risk of cardiovascular disease in MS, especially ischemic stroke and myocardial infarction, directly associated with abnormal platelet function redirected to their prothrombotic activity [14–20]. Chronic inflammation and massive ROS/RNS production may be the main cause of excessive platelet activation in MS [21]. Platelet functioning strictly depends on the activity of prooxidative processes and their current red-ox state. Platelet aggregation could be induced by H_2_O_2_ (a source of hydroxyl radicals), which suggests that ROS may act as “second messengers” during the initial phase of the platelet activation process [22]. Blood platelets have an inherent ability to produce ROS by various pathways, including as a by-product of the respiratory pathway [23]. Despite the lack of a cell nucleus, platelets contain basic cellular organelles, including numerous mitochondria (from 5 to 8), and maintain an active metabolism [24]. These tiny size cells arrive first at sites of vascular injury and can be seen as substantial players in neurodegenerative diseases. The aim of the present study was to assess the oxidative/nitrative modifications of blood platelet proteins in the course of SP MS and correlation of their levels with the degree of psychophysical disability of patients.

## 2. Materials and Methods

### 2.1. Demographic and Clinical Characteristics

The 110 patients with SP MS and 110 healthy volunteers were included in the study. All patients with SP MS were diagnosed according to the revised McDonald criteria [25]. Patients were observed for one year prior to the commencement of the study, and the clinical parameters of patients with SP MS are included: mean age 48.2 ± 15.2 years, mean disease duration 14.3 ± 8.3 years, EDSS 5.5 ± 1.8, and BDI 9.6 ± 4.6. The healthy volunteers did not take any medications and had never been diagnosed with MS or other neurodegenerative disease and any chronic inflammation. The mean platelet counts in SP MS patients and controls were within the reference range (150 − 450 × 10^3^/*μ*l). The control group and SP MS patients were matched by the number, age, and sex in Table 1.

The EDSS scale is a method for quantifying the degree of disability in the course of MS. The scale range of EDSS is from 0 (no disability) to 10 (death due to MS) [26, 27]. BDI is a psychometric test for measuring the severity of depression. The presence of depression is observed when the BDI score is above 13, and major depression when the score is ≥30 [28].

Blood samples from SP MS patients were delivered from Neurological Rehabilitation Division III General Hospital in Lodz, Poland, and those from healthy volunteers were collected at Laboratory Diagnostics Center in Lodz, Poland. The protocol and all procedures were conducted according to the Helsinki declaration and were approved by the Ethics Committee of the Faculty of Biology and Environmental Protection of University of Lodz, Poland No.5/KBBN-UŁ/II/2013.

### 2.2. Isolation of Blood Platelets

The human blood samples, which were taken from a peripheral vein between 8 and 9 a.m., were kept in CPDA-1 (citrate phosphate dextrose adenine-1). Blood platelets were isolated from freshly collected blood by using a modified method of BSA-Sepharose 2B gel-filtration described by Walkowiak et al. [29], and resuspended in modified Tyrode's (Ca^+2^/Mg^+2^) free buffer (127 mM NaCl, 2.7 mM KCl, 0.5 mM NaH_2_PO_4_, 12 mM NaHCO_3_, 5 mM HEPES, 5.6 mM glucose, pH 7.4). The number of platelets was determined based on the photometric method according to Walkowiak et al. [30] and were routinely diluted to obtain 2 × 10^8^ platelets/ml. Platelet suspensions for the determination of 3-NT and carbonyl group were diluted in lysis buffer 1 : 1 (2% Triton X-100, 100 mM EDTA, 0.1 M Tris-HCl, pH 7.4) and stored in -80°C until analysis.

### 2.3. Measurement of the Level of Carbonyl Groups by the ELISA Method

The detection of carbonyl groups, estimated as adducts of 2,4-dinitrophenylhydrazine (DNPH), was carried out according to the ELISA method described by Buss et al. [31] and modified by Almadari et al. [32]. The microplates were incubated with a blocking buffer, overnight at 4°C, to block any nonspecific binding. Following three washes with 300 *μ*l PBS, the frozen blood platelet proteins (-80°C) were reacted with substrate DNPH. After incubation for 45 minutes at room temperature in the dark, all wells were washed 5 times with 300 *μ*l PBS : ethanol (1 : 1, *v*/*v*) and with 300 *μ*l PBS. The carbonyl groups were detected by the first anti-DNP antibodies and then by the second antibody conjugated with horseradish peroxidase. The level of carbonyl groups was determined spectrophotometrically (at *λ* = 316 *nm*) according to the Levine et al. [33]. To confirm the linearity of the ELISA method, the standard curve was prepared using oxidized albumin, which expressed nmol carbonyl groups/mg of albumin.

### 2.4. Determination of 3-Nitrotyrosinethe Level by the Competitive ELISA Method

The detection of 3-NT in the frozen human blood platelet proteins (-80°C) was performed according to the Khan et al. [34] using the competitive ELISA test. The concentration of 3-NT was assessed based on the standard curve, drawn up of the 3-NT containing fibrinogen (3-NT-Fg). The human fibrinogen was treated with the peroxynitrite at the final concentration of 1 mM to obtain the 3-NT-Fg. The amount of 3-NT-Fg was spectrophotometrically determined at the *λ* = 430 nm (*ε* = 4,400 M^−1^ cm^−1^) with a plate reader. After the spectrophotometric measurement, the nitro-fibrinogen was used to prepare the standard curve, ranging from 10 to 1000 nM/l of 3-NT-Fg equivalent.

### 2.5. The Sulfhydryl Group (-SH) Measurement

The total concentration of protein -SH groups was denoted by the spectrophotometric method originally described by Ando and Steiner [35]. According to this method, free thiol groups react with the 5,5′-dithiobis-2-nitrobenzonic acid (DTNB) and the colored thiols are formed. To the frozen blood platelet samples (-80°C), 500 *μ*l of protein precipitating solution buffer was added (0.85% H_3_PO_4_, 0.2% EDTA, 30% NaCl). Acid-insoluble (proteins) platelet fractions were separated according to Ando and Steiner [35]. All samples were double frozen and thawed and then centrifuged (3000 g, 20 min.). To the pellet (acid-precipitable fraction), 3 ml of 10% SDS was added. To the obtained pellet, 3.2 ml of 0.32 M Na_2_HPO_4_, 250 *μ*l of 4 mM DNTB in 1% sodium citrate was added. All samples were incubated for 30 minutes at room temperature. The concentration of free sulfhydryl groups was spectrophotometrically estimated at *λ* = 412 nm and was expressed as nmol/mg platelet proteins.

### 2.6. Measurement of NADPH Oxidase 1 Concentration

The concentration of NOX-1 in a frozen suspension of blood platelets (-80°C) obtained from patients with SP MS and healthy volunteers was determined using Human NOX-1 ELISA Kit (Fine Test, Wuhan, China). In this kit, the biotin conjugated anti-Human NOX-1 antibody was used to detect antibodies. HRP- (horseradish peroxidase-) streptavidin was added, and then, 3,3′,5,5′-tetramethylbenzidine (TMB) substrate was used to visualize the HRP enzymatic reaction producing a blue color product that changed yellow after adding an acidic stop solution. The level of NOX-1 was determined spectrophotometrically at *λ* = 450 *nm*. The results are presented in ng/ml of platelet suspension, based on a standard calibration curve.

### 2.7. Analysis of MMP in Blood Platelets

MMP was determined by the fluorescent dye JC-1 (Molecular Probes, Eugene, OR, USA) [36]. In undamaged mitochondria, JC-1 dye accumulates in large amounts in a hyperpolarized membrane, where it forms aggregates that emit red fluorescence (*λ*ex = 530 nm, *λ*em = 590 nm). During depolarization and permeation of the mitochondrial membrane, there is the breakdown of aggregates into monomers emitting green fluorescence, similar to that of fluorescein (*λ*ex = 485 nm, *λ*em = 538 nm). The red to green fluorescence intensity ratio (reflecting the level of mitochondrial membrane damage) is only dependent on the membrane potential, and there are no other factors such as shape, mitochondrial size, or density that can influence the single-component fluorescence signals. The human blood platelets freshly isolated by BSA-Sepharose 2B gel-filtration were suspended in modified Tyrode's Ca^2+^/Mg^2+^ free buffer (2 × 10^8^ platelets/ml). A  stock  solution  of JC-of 1500 *μ*M was prepared in dimethyl sulfoxide (DMSO) and added into the platelet suspension to a final concentration of 5 *μ*M. The platelet suspension with JC-1 and without a dye (control) was preincubated on black 96-well plates with a transparent bottom (Greiner Bio-One, Monroe, NC, US) and incubated at 37°C for 30 min. The fluorescence was measured immediately after incubation using the Bio-Tek Synergy HT Microplate Reader (Bio-Tek Instruments, Winooski, VT, US).

### 2.8. Measurement of Intracellular ROS Levels

The level of intracellular ROS in blood platelets was measured using the red-ox-sensitive fluorescent dye-DCFH-DA (Molecular Probes, Eugene, OR, USA) [37]. The freshly isolated blood platelet samples were preincubated with 5 *μ*M of DCFH-DA (prepared in Tyrode's Ca^2+^/Mg^2+^ free buffer), at 37°C for 30 min. Fluorescence was measured at an excitation wavelength of 480 nm and an emission wavelength of 510 nm, using a Bio-Tek Synergy HT Microplate Reader (Bio-Tek Instruments, Winooski, VT, USA). Results are expressed as the level of DCF-DA fluorescence ± SD, which corresponds to the amount of ROS produced in platelets in both SP MS and control groups.

### 2.9. Isolation of RNA and Reverse Transcription (RT-PCR)

Total RNA was isolated from frozen (-80°C) blood platelets suspended in RNAlater solution (Invitrogen, Carlsbad, CA, USA) using the Isolate II RNA Mini Kit (Bioline, London, GB) following the manufacturer's instructions. RNA concentration was determined by spectrophotometric measurement of absorbance at 260 nm, and purity was calculated at a ratio of A260/A280 with a Bio-Tek Synergy HT Microplate Reader (BioTek Instruments, Inc., Winooski, VT, USA). Total RNA (1 *μ*g) was reverse transcribed into cDNA with a Maxima First Strand cDNA Synthesis Kit for RT-qPCR (Thermo Fisher Scientific Waltham, MA, USA). All steps were performed according to the manufacturer's recommendations.

### 2.10. Expression of mRNA by RT-qPCR Method

Quantitative Real-Time PCR (RT-qPCR) was performed to determine relative expression of mRNA using the following TaqMan probes: Hs02596864_g1 for the *MTCO-1* gene (mitochondrial encoded cytochrome C oxidase I), Hs02786624_g1 for the *GAPDH* gene (glyceraldehyde-3-phosphate dehydrogenase), and Hs99999901_s1 as an endogenous control (the human 18SrRNA gene), sourced from Life Technologies, Carlsbad, CA, USA. RT-qPCR analyses were performed using a CFX96 Real-Time PCR system (Bio-Rad Laboratories, Hercules, CA, USA), with a TaqMan Universal Master Mix II without UNG (Life Technologies, Carlsbad, CA, USA). All procedures were performed according to the manufacturers' protocols. The Ct values were calculated automatically, and the analyses performed using CFX Manager™ Software (version 3.1). Relative expressions of mRNA for the studied genes were calculated using the 2^−*Δ*Ct^ method, where ΔCt = Ct_target gene_ − Ct_18SrRNA_.

### 2.11. Statistical Analysis

StatsDirect statistical software V.2.7.2. was used to perform the statistical analysis. All values were expressed as a mean ± SD. The Shapiro-Wilk test was used to analyze the normality of the distribution of results. The significance of the differences was analyzed depending on their normality by using the unpaired Student-*t* test (for data with normal distribution) or Mann–Whitney *U* test (for data with abnormal distribution). Spearman's rank correlation was used for correlation analysis between oxidative stress biomarkers and clinical parameters: EDSS and BDI. A level of *p* < 0.05 was accepted as statistically significant.

## 3. Results

### 3.1. The Level of Oxidative Stress Markers and Their Correlation with BDI and EDSS Scales

**Figure 1:**
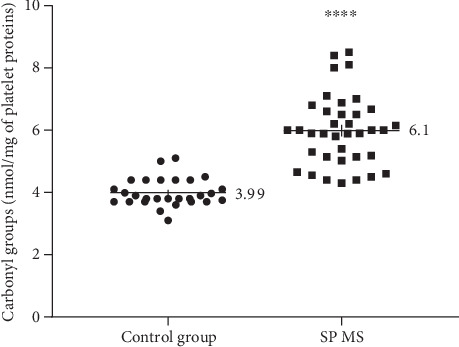
The concentration of carbonyl groups in blood platelets, determined immediately after isolation from whole, untreated blood in SP MS patients (*n* = 40) and the control group (*n* = 40). The results are expressed as values obtained for individual subjects (nmol/mg of platelet proteins) with the mean value ± SD. Statistical analysis was performed using the unpaired Student-*t*, ^∗∗∗∗^*p* < 0.0001.

**Figure 2:**
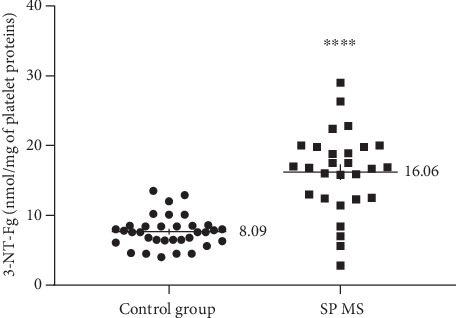
The concentration of 3-NT in blood platelets, determined immediately after isolation from whole, untreated blood in SP MS patients (*n* = 40) and control group (*n* = 40). The results are expressed as values obtained for individual subjects (nmol/mg of platelet proteins) with the mean value ± SD. Statistical analysis was performed using the unpaired Student-*t*, ^∗∗∗∗^*p* < 0.0001.

**Figure 3:**
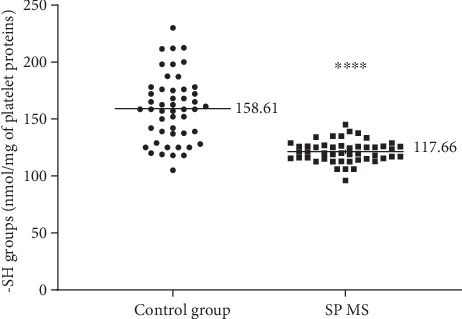
The concentration of –SH groups in blood platelets, determined immediately after isolation from whole, untreated blood in SP MS patients (*n* = 50) and the control group (*n* = 50). The results are expressed as values obtained for individual subjects (nmol/mg of platelet proteins) with the mean value ± SD. Statistical analysis was performed using the unpaired Student-*t*, ^∗∗∗∗^*p* < 0.0001.

**Figure 4:**
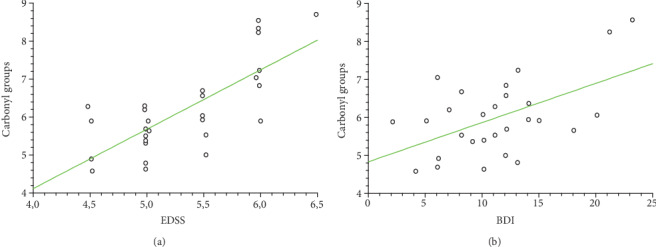
Regression plots of carbonyl group levels in platelet proteins obtained from SP MS patients and EDSS (a), BDI (b) scales.

**Figure 5:**
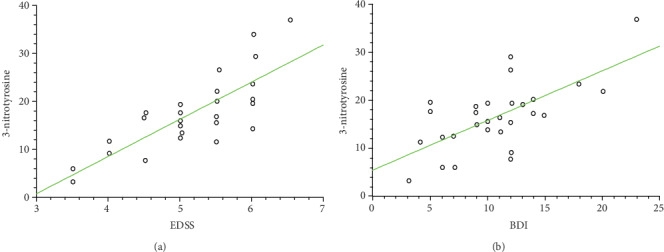
Regression plots of 3-NT levels in platelet proteins obtained from SP MS patients and EDSS (a), BDI (b) scales.

Our data demonstrate the high level of oxidative/nitrative damages in platelet proteins obtained from SP MS patients. In our studies on SP MS patients, we have shown the statistically significant increased level of carbonyl groups (6.1 nmol/mg in SP MS vs. 3.99 nmol/mg in control, *p* < 0.0001) (Figure 1), 3-NT (16.06 nmol/mg in SP MS vs. 8.09 nmol/mg in control, *p* < 0.0001) (Figure 2), and the statistically significant decreased level of thiol groups (117.66 nmol/mg in SP MS vs. 158.61 nmol/mg in control, *p* < 0.0001 (Figure 3) compared to the control group. Moreover, we established the positive correlation between the level of carbonyl groups (Figures 4(a)and 4(b); Table 2), 3-NT (Figures 5(a) and 5(b); Table 2) and EDSS or BDI scales.

### 3.2. Measurement of Intracellular ROS Level

**Figure 6:**
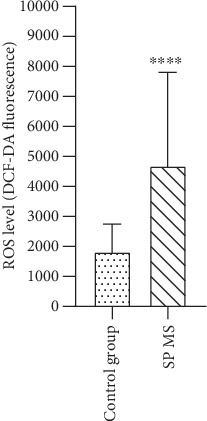
The level of intracellular ROS generated in blood platelets. ROS production measured as intensity of DCF fluorescence in SP MS patients (*n* = 30) and control group (*n* = 30). Statistical analysis was performed using the Mann–Whitney *U* test. The results are expressed as the mean value of DCF-DA fluorescence ± SD, ^∗∗∗∗^*p* < 0.0001.

The next step in our studies was a measurement of intracellular concentration of ROS generated in blood platelets. The results obtained showed that the level of ROS in untreated platelets from SP MS patients was 2.5-fold higher compared to that of control (DCF-DA fluorescence was 4.689 in SP MS vs. 1.816 in control, *p* < 0.0001) (Figure 6).

### 3.3. Analysis of Changes in the MMP in Blood Platelets Using the JC-1 Method

**Figure 7:**
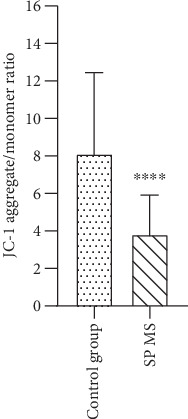
Changes in MMP (*ΔΨ*m) in blood platelets. MMP is expressed as a ratio of JC-1 aggregates (530 nm/590 nm) to JC-1 monomers (485 nm/538 nm), as quantified with a fluorescent plate reader after JC-1 staining in blood platelets obtained from SP MS patients (*n* = 30) and the control group (*n* = 30). Statistical analysis was performed using the Mann–Whitney *U* test. The results are shown as the mean value of fluorescence ratio (590 nm/538 nm) ± SD, ^∗∗∗∗^*p* < 0.0001.

JC-1 dye can be used both as qualitative (considering the shift from green to red fluorescence emission) and quantitative (considering only the pure fluorescence intensity) measure of MMP. The ratio of the fluorescence of JC-1 aggregates to JC-1 monomers (590 nm/538 nm) reflects the level of membrane damage to cell mitochondria. The results we obtained indicate an over 1.5-fold lower (*p* < 0.001) ratio of aggregates to monomers of the JC-1 dye in platelets from people with SP MS compared to the control group (8.1 vs. 3.8, respectively) (Figure 7). The decrease in the aggregated form of the JC-1 dye indicates the depolarization and permeability of the platelet mitochondrial membrane in SP MS relative to normal platelets.

### 3.4. The Expression of Enzymes Involved in the Red-Ox Status

**Figure 8:**
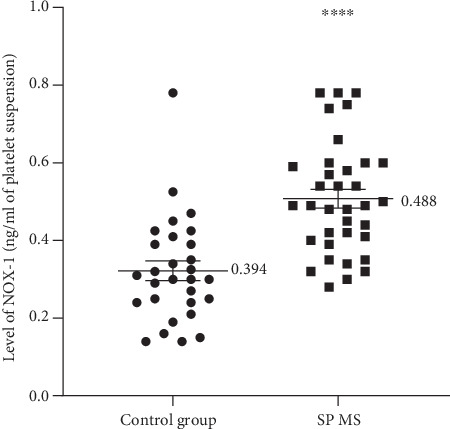
The concentration of NOX-1 in blood platelets, determined immediately after isolation from whole blood untreated. The comparison of NOX-1 expression level (ng/ml) measured in blood platelets in SP MS patients (*n* = 30) and control group (*n* = 30). The results are expressed as values obtained for individual subjects with the mean value ± SD. Statistical analysis was performed using the Mann–Whitney *U* test, ^∗∗∗∗^*p* < 0.0001.

In another set of experiments, we found that the level of NOX-1 was significantly higher by about 30% *p* < 0.0001 in platelets from SP MS patients than in the control group (0.49 ng/ml vs. 0.39 ng/ml, respectively) (Figure 8).

**Figure 9:**
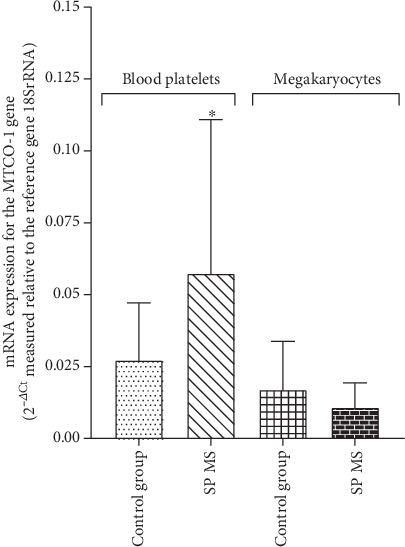
The mRNA level of *MTCO-1* gene in blood platelets and megakaryocytes. Relative expression of mRNA for the *MTCO-1* gene in platelets and megakaryocytes from patients with SP MS (*n* = 45) and control group (*n* = 45). Statistical analysis was performed using the Mann–Whitney *U* test. The results are expressed as the mean value of 2^−ΔCt^ ± SD (according to the reference gene–18SrRNA), ^∗^*p* < 0.05.

Analysis of the relative expression of mRNA for the *MTCO-1* gene in blood platelets showed a more than 2-fold statistically significant (*p* < 0.05) increase in the level of transcripts for the *MTCO-1* gene in SP MS, compared to the control group (2^-*Δ*Ct^ was 0.057 in SP MS vs. 0.027 in control). Analogous analysis for megakaryocytes presented no statistically significant differences between the compared groups (2^-*Δ*Ct^ was 0.017 in SP MS vs. 0.011 in control, *p* > 0.05) (Figure 9).

**Figure 10:**
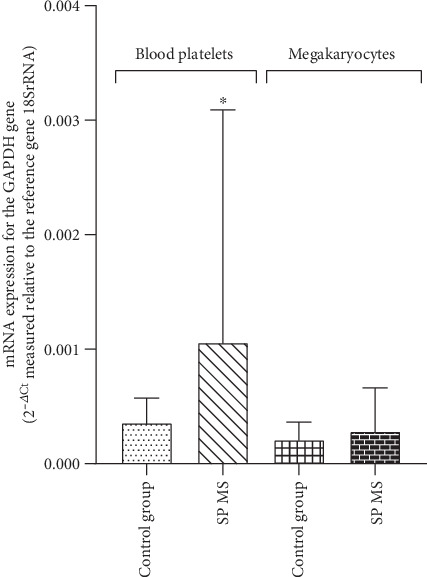
The mRNA level of *GAPDH* gene in blood platelets and megakaryocytes. The relative expression of mRNA for the *GAPDH* gene in platelets and megakaryocytes from patients with SP MS (*n* = 45) and control group (*n* = 45). Statistical analysis was performed using the Mann–Whitney *U* test. The results are expressed as the mean value of 2^−ΔCt^ ± SD (according to the reference gene–18SrRNA), ^∗^*p* < 0.05.

Measurement of the relative mRNA expression of *GAPDH* gene in platelets showed an approximately 2.5-fold statistically significant (*p* < 0.05) increase in the level of transcripts for *GAPDH* gene in SP MS, in comparison to the control group (2^-*Δ*Ct^ was 0.0011 in SP MS vs. 0.0004 in control). However, in the case of megakaryocytes, also for these transcripts, no statistically significant differences were found between the compared groups (2^-*Δ*Ct^ was 0.00028 in SP MS vs. 0.00021 in control, *p* > 0.05) (Figure 10).

## 4. Discussion

Oxidative stress in patients with MS is associated with an increase damage of myelin and axonal that may lead to the appearance of clinical symptoms [11, 38–40]. ROS/RNS generation in the course of MS alters brain plasticity and through oxidative damage causes disturbances in neurotransmission and formation of new cells [41]. In chronic inflammatory diseases, such as MS, antioxidant defenses are eclipsed, which leads to oxidative stress [42]. The latest research conducted by Siotto et al. has shown that in relapsing-remitting multiple sclerosis (RR MS) patients with low disability (EDSS near 1.0) and short duration of the disease (approximately 2 years), the oxidative stress status is elevated. It is revealed by low levels of total antioxidant status (TAS) and high levels of total plasma hydroperoxides. In RR MS, the neurodegeneration rate is usually relatively low, but there is a strong oxidative imbalance associated with the development of the inflammatory process and autoimmunity [43].

Demyelination and axonal destruction, which are both the pathological hallmarks of MS, are mainly caused by overproduction of ROS and RNS generated by invading inflammatory cells, as well as resident CNS cells. Generation of ROS/RNS (O_2_^−·^, OH^−^, H_2_O_2_, NO, and ONOO^−^) contributes to several mechanisms underlying the pathogenesis of MS lesions (oxidation/nitration of proteins, lipid peroxidation, damage to nucleic acids, enzyme inhibition, and activation of programmed cell death pathway) [44]. High levels of NO, ONOO^−^, and O_2_^−·^ have all been demonstrated in spinal fluid from patients with MS [45]. Myelin, which surrounds the axons, consists of 30% proteins and 70% lipids and is the major target of a ROS/RNS attack in MS [46].

ROS/RNS generation is responsible for demyelination and neurodegeneration in MS, but it is also likely to cause cardiovascular disorders in MS associated with the impaired response of blood platelets [47]. Currently, there is relatively little information about the functioning of blood platelets during neurodegeneration and knowledge about their participation in these diseases is still neglected and poorly understood. However, available clinical data have confirmed the elevated activation of circulating platelets in MS [48]. Our previous study revealed an increase in the prothrombotic and proinflammatory properties of platelets [11, 49, 50]. In neurobiological studies, blood platelets are regarded as a cellular model for the analysis of metabolic pathways related to the pathogenesis of MS and the regulation of oxidative stress [49, 51]. Oxidative/nitrative species are implicated in the regulation of platelet function, and during platelet activation, in the receptor-mediated signalling pathways, they may be produced as the “second messengers' [52]. In the following years, the participation of several platelet-derived ROS in MS, including O_2_^−·^, OH^−^, and H_2_O_2_, after stimulation with typical agonists was reported [53]. ROS production in activated blood platelets is mainly dependent on the activity of NOX and xanthine oxidase, an enzymatic cascade of arachidonic acid metabolized *via* COX, glutathione (GSH) cycle, and metabolism of phosphoinositides [54].

Two independent research groups, Begonja et al. [55] and Krötz et al. [56] have proven that during platelet activation, mainly O_2_^−·^ is produced. They have shown that NOX-dependent platelet O_2_^−·^ formation increases platelet aggregation by stimulation of the phospholipase A2-dependent arachidonic acid released from the platelet membrane. Our previous studies indicated a positive correlation between the level of O_2_^−·^ in platelets and their amplified activation expressed as elevated adhesion and aggregation in SP MS [11]. Since the presence of NOX-1 in the cell membrane of blood platelets is one of the major regulators of ROS production, our current studies were designed to demonstrate the difference in the expression level of NOX-1 between platelets from SP MS patients and healthy controls. Our findings have confirmed that the platelet expression of NOX-1 in SP MS patients is significantly elevated (by approximately 30%) compared to its level in platelets from healthy subjects (Figure 8). The elevated level of ROS generated within platelets in SP MS has been demonstrated by us using a fluorescent method with DCFH-DA dye, which enters through the cell membrane and is enzymatically hydrolyzed with intracellular esterases to nonfluorescent DCFH, which is then oxidized to highly fluorescent dichlorofluorescein (DCF) in the presence of intracellular ROS [54]. That analysis showed that the level of intracellular ROS in blood platelets from SP MS patients is much higher than in the control group (*p* < 0.0001) (Figure 6).

In blood platelets devoid of the nucleus, the proteins are the main target of ROS/RNS [8]. Exposure of proteins to ROS/RNS can cause major physical changes in protein structure [47]. The oxidative/nitrative damage of proteins leads to the peptide backbone cleavage, cross-linking and/or modifications of the side chain of every amino acid [57]. The great number of protein injuries is irreparable, and oxidative/nitrative changes of protein structure have functional consequences, such as inhibition of enzymatic activities, a misfolding, the increased ability of proteins to aggregation and proteolysis, and altered immunogenicity [58]. In our studies, the oxidative damage of platelet proteins has been demonstrated as a significantly increased level of carbonyl groups in SP MS patients (Figure 1). The results obtained are in line with other reports indicating an elevated level of carbonyl proteins in neurodegenerative disease. Bizzozero et al. found an increased amount of protein carbonyls in the brain white and grey matter of patients with MS [59]. Rommer et al. also showed a raised level of carbonyl groups in cerebrospinal fluid in MS patients compared to the control group [60].

Certain studies have suggested that there is a link between depression severity and the progression of the disease. Major Depressive Disorder (MDD) is extraordinarily common in MS (prevalence of depression 15-30%). MDD lifetime prevalence rates in patients with MS are close to 50%, i.e., three times higher than in the general population. Depression is more common among patients with MS than other chronic illnesses, including other coexisting neurologic disorders [61, 62]. In our study, we have shown the positive correlation between the intensity of carbonyl group formation in blood platelet proteins obtained from SP MS patients and clinical parameters EDSS (Figure 4(a)) or BDI (Figure 4(b)). Our findings indicate that the level of carbonyl groups might be a useful biomarker expressing the progression of neurodegenerative processes caused by ROS/RNS.

NO is a chemical compound that contributes to the regulation of blood flow and participates in some synaptic transmissions. High levels of NO, ONOO^−^ and O_2_^−•^ have all been demonstrated in spinal fluid from patients with MS [45, 63]. The studies of Bö et al. indicate that NO has a role in the pathogenesis of demyelinating MS lesions because of the markedly elevated human inducible nitric oxide synthase (iNOS) present in tissue sections from MS [64]. In the fast reaction between NO and O_2_^−·^, short survival ROS and RNS capable of inducing oxidative/nitrative changes in a wide variety of biomolecules are produced. Among them, the highest activity exhibits ONOO^−^ [65]. ONOO^−^ is a highly reactive compound that modifies tyrosine residues resulting in protein nitration, expressed as the 3-NT [66, 67]. It is known that 3-NT is a strong NO-derived oxidant and the earliest detectable biomarker found in the plaques of the brain from patients suffering from MS and other neurodegenerative disorders [68, 69]. In our studies, the elevated level of 3-NT in platelet proteins obtained from SP MS patients, compared to the control group, was observed (Figure 2). We also revealed the positive correlation between the increased level of 3-NT and EDSS or BDI in SP MS patients (Figures 5(a) and 5(b), respectively). Our findings are consistent with the report of Jack et al., who detected the raised level of 3-NT in active demyelinated lesions in MS [70]. Thus, we emphasize the importance of platelets in the pathogenesis of MS, in inflammatory lesions, where red-ox balance is disturbed.

Individual proteins can display different susceptibilities to an oxidative attack, which is linked to the distribution of -SH groups. The thiol-disulphide homeostasis is important in antioxidant defense, detoxification, apoptosis, signal transduction, regulation of enzymatic activity, and transcription and intracellular signalling mechanisms [71]. Mitosek-Szewczyk et al. showed a reduced level of protein -SH groups in cerebrospinal fluid and serum delivered from MS patients [72]. Our blood platelet studies also confirm the strong oxidation of protein thiol groups in SP MS patients (Figure 3). However, the correlations between the level of -SH groups and EDSS as well as BDI scale have not been observed.

The rate of ADP turnover in blood platelets is even greater than in resting mammalian muscle, suggesting an essential role for mitochondria (and red-ox signalling) in platelet function. Furthermore, energy demand escalates during the platelet activation and the complex signalling cascade that regulates adhesion, aggregation, and secretion processes [73]. This incremental energy consumption is one of the main determinants of platelet function. Mitochondria encode functionally important subunits of the mitochondrial respiratory chain complexes, located in the inner mitochondrial membrane that consists of four complexes (complexes I–IV) and complex V (ATP synthase) [74]. Cytochrome c oxidase (complex IV) is the last and key enzyme of the electron transport chain in the oxidative phosphorylation process, encoded by the mitochondrial *MTCO-1* gene. This terminal complex is where over 90% of cell oxygen is consumed. Cytochrome c oxidase is the component of the respiratory chain that catalyzes the reduction of O_2_ to H_2_O. [75]. The MMP (*ΔΨ*m) generated by proton pumps is an essential component in the process of energy storage during oxidative phosphorylation [76]. The decrease of *ΔΨ*m may be a signal of loss of cell viability leading to various pathologies. GAPDH is a multifunctional protein that also mediates cell death under oxidative stress. In the largest amounts, the enzyme is present in the cytoplasm, but part of the enzyme is also found in mitochondria [77]. Recent studies have shown that GAPDH has a significant role in ATP production and autoimmune response-induced neuroaxonal pathological changes in MS. GAPDH as a membrane protein is involved in energy generation, polymerization of tubulin into microtubules, and the control of protein synthesis in the endoplasmic reticulum [78]. GAPDH is extremely sensitive to oxidative stress modifications and damage, and, it is even considered to be one of the main cellular targets for ROS and RNS [79]. However, there are a lot of studies in which *GAPDH* was used as housekeeping gene (reference gene) and is shown to be the most stable gene in platelets used in mRNA expression studies [80, 81]. Surprisingly, in our studies, we showed that the amount of mRNA transcripts for the *GAPDH* gene in SP MS patients is pathologically increased in comparison to the control group (Figure 10). Our findings have suggested the presence of spontaneous mitochondrial damage in SP MS, which is also confirmed by the decrease in the expression of transcripts for the *MTCO-1* gene in SP MS (Figure 9) and the significant depolarization of platelet mitochondrial membrane in SP MS patients (Figure 7), compared to the control group.

## 5. Conclusion

We suggest that oxidative/nitrative damage of platelet proteins is implicated in the pathophysiology of MS, reflecting disease status expressed as a degree on the EDSS and BDI scales. The use of biomarkers such as 3-NT or carbonyl groups may improve the sensitivity and specificity of the measurement of the risk factor, namely, oxidative stress. We strongly recommend the introduction of antiplatelet and antioxidative therapy in the future treatment of MS.

## Figures and Tables

**Table 1:** The characteristics of control and study group (SP MS).

	Control group (*n* = 110)	SP MS (*n* = 110)
Mean age (years)	48.7 ± 12.21	48.2 ± 15.2
Gender:		
Male	43	43
Female	67	67
BMI (kg/m^2^)	21.9 ± 4.85	21.1 ± 9.7
EDSS	NA	5.5 ± 1.8
BDI	NA	9.6 ± 4.6
Mean disease duration (years)	NA	14.3 ± 8.3

Abbreviations: EDSS: Expanded Disability Status Scale; BDI: Beck's Depression Inventory; SP MS: secondary progressive multiple sclerosis; NA: not applicable; BMI: body mass index.

**Table 2:** Correlation coefficient values obtained for oxidative stress biomarkers and EDSS and BDI scales. Correlation was analyzed using Spearman's rank correlation method. Table consists of Spearman's rank correlation coefficient (Rho) and probability of correlation (*p*).

EDSS		
	Carbonyl groups	3-NT
Spearman's rank correlation (rho)	0.706381	0.723329
Probability of correlation	*p* = 0.0001	*p* = 0.0001
BDI		
	Carbonyl groups	3-NT
Spearman's rank correlation (rho)	0.357093	0.417139
Probability of correlation	*p* = 0.02	*p* = 0.01

## Data Availability

The datasets used and analyzed during the current study are available from the corresponding author on request.
